# Supplementary material for: The genome sequence of the Four-spotted Footman moth, Lithosia quadra (Linnaeus, 1758)
Source: Wellcome Open Res. 2025 Mar 19;10:146. [Version 1] doi: 10.12688/wellcomeopenres.23788.1 (PMC12038344; doi:10.12688/wellcomeopenres.23788.1)
Supplement: Peer review history

# Reviewer report: Reviewer response for version 1

**DOI:** 10.21956/wellcomeopenres.26237.r121846

**Published:** 2025-05-03

**Authors:** Arun Arumugaperumal

**Affiliations:** 1 Rajalakshmi Engineering College, Chennai, India

This is the first report of the genome sequence of Lithosia quadra, also known as the Four-spotted Footman moth. The authors have used PacBio long read sequencing and Hi-C data to arrive at a high quality assembly. The genome size obtained was 456.7 Mb spread in 31 chromosomes. The mitogenome was also assembled with size 15.38 kb. 

 As the other reviewer pointed out the sex of the sample was mentioned wrong in the manuscript. But on checking the biosample accession SAMEA112775073, the sex of the sample was recorded as 'NOT COLLECTED'. The authors need to change this metadata also.

 A BUSCO score of 98.8% indicates the good quality of the genome. The genome data can be indexed.

Are sufficient details of methods and materials provided to allow replication by others?

Yes

Is the rationale for creating the dataset(s) clearly described?

Yes

Are the datasets clearly presented in a useable and accessible format?

Yes

Are the protocols appropriate and is the work technically sound?

Yes

Reviewer Expertise:

Bioinformatics; Genomics

I confirm that I have read this submission and believe that I have an appropriate level of expertise to confirm that it is of an acceptable scientific standard.

# Reviewer report: Reviewer response for version 1

**DOI:** 10.21956/wellcomeopenres.26237.r121847

**Published:** 2025-04-28

**Authors:** Sivasankaran Kuppusamy

**Affiliations:** 1 Loyola College, Chennai, India

The authors have done the chromosome-level genome assembly of *Lithosia quadra *(Linnaeus, 1758). Authors have used the appropriate methods for the DNA isolation, purification and library preparation for sequencing. Appropriate software’s were used for assembly and sequence annotation. They observed a 456.17 Mb of genome sequence of the species. They have assigned synteny Z chromosome with the genome of *Nudaria mundana*.

**Comments on the manuscript**

 The first sentence of the Background can be modified as “ *Lithosia quadra* (Linnaeus, 1758) or the Four-spotted Footman moth belonging to the family Erebidae”.

 The last sentence of the first paragraph “With a wingspan of 35-55 mm....” can be deleted.

 Last paragraph of the Background can be modified as “Here we present a chromosome-level genome assembly of *L. quadra*………..

 Throughout the manuscript, the authors consistently used the genus name *Lithosia quadra* in its full form. If possible, use the full genus name at first, and later to shorten it to *L. quadra*

**

 Authors have sequenced the genome’s total length of 112.29 Gb using three different sequencing platforms. Though authors assembled and received 456.27 Mb  genome in size but haven’t observed the protein-coding genes, non-coding genes and gene transcripts.  What is the reason?

 Authors have mentioned the sequence was done based on a male specimen in the Abstract, Background and Genome sequence report. But in the Methods authors have mentioned “An adult female *Lithosia quadra* was collected from Wytham Woods Oxfordshire”. I think there is an error. Authors can change it. 

 Above all, I confirm that the manuscript meets the necessary scientific standard and is suitable for indexing

Are sufficient details of methods and materials provided to allow replication by others?

Yes

Is the rationale for creating the dataset(s) clearly described?

Yes

Are the datasets clearly presented in a useable and accessible format?

Yes

Are the protocols appropriate and is the work technically sound?

Yes

Reviewer Expertise:

Phylogenetic analysis of Moths using mitogenome sequence

I confirm that I have read this submission and believe that I have an appropriate level of expertise to confirm that it is of an acceptable scientific standard.

# Reviewer report: Reviewer response for version 1

**DOI:** 10.21956/wellcomeopenres.26237.r121085

**Published:** 2025-04-19

**Authors:** Yu-Feng Huang

**Affiliations:** 1 National Chung Hsing University, Taichung, Taiwan

This manuscript presents a high-quality, chromosomal-level genome assembly of Lithosia quadra, the Four-spotted Footman moth, a lichen-feeding Erebidae species with widespread distribution across the Palearctic region. Generated as part of the Darwin Tree of Life (DToL) initiative, the assembly is based on PacBio HiFi long-read sequencing and Hi-C scaffolding. The final genome spans 456.27 Mb, with 99.92% of the assembly placed on 31 chromosomal pseudomolecules, including the Z chromosome. The authors also assembled the 15.38 kb mitochondrial genome. Quality metrics are excellent, with a primary haplotype consensus QV of 66.7, a scaffold N50 of 16.41 Mb, and a BUSCO completeness score of 98.8% (based on the lepidoptera_odb10 dataset). The assembly meets the Earth BioGenome Project’s reference standard (6.C.Q66).

 The technical and methodological execution of the project is of high standard. Sequencing, assembly, curation, and evaluation are thoroughly documented, supported by open-access tools and interactive visualizations. The manuscript’s value lies in contributing to the broader effort of cataloguing biodiversity genomics within Lepidoptera, and Erebidae in particular. However, several areas of improvement are necessary to elevate the manuscript’s scientific utility and clarity.

 Major Concerns

1. Haplotype Representation and Diversity
  While both primary and alternate assemblies are deposited, the manuscript lacks discussion on phased regions or heterozygosity. With an estimated 2.02% heterozygosity, the authors should clarify whether any haplotype-resolved analysis was attempted or if structural differences were observed.
2. Unplaced Scaffolds
  The assembly claims 99.92% chromosomal placement, yet there is no description of the remaining 0.08%. Are these repeats, contamination, or unresolved genomic regions? A short explanation would improve transparency.
3. Mitochondrial Genome Representation
  The mitochondrial genome is included alongside nuclear chromosomes in Table 3, which could be confusing due to its much smaller size. It should be reported separately, as is standard in genomic publications. Also, the mitochondrial genome length is given in floating-point (15.38 kb) in multiple places and should be reported as the full integer value for accuracy.

Minor Concerns

- A comparison of the presented assembly quality (e.g., QV, k-mer completeness) with other Lepidoptera genomes would provide helpful context.
- The Methods section is highly technical; a concise overview or flow diagram could help general readers.

Are sufficient details of methods and materials provided to allow replication by others?

Yes

Is the rationale for creating the dataset(s) clearly described?

Yes

Are the datasets clearly presented in a useable and accessible format?

Yes

Are the protocols appropriate and is the work technically sound?

Partly

Reviewer Expertise:

Bioinformatics, Genome assembly and annotation, Structural bioinformatics, Cancer biology

I confirm that I have read this submission and believe that I have an appropriate level of expertise to confirm that it is of an acceptable scientific standard, however I have significant reservations, as outlined above.

# Reviewer report: Reviewer response for version 1

**DOI:** 10.21956/wellcomeopenres.26237.r121843

**Published:** 2025-04-08

**Authors:** Axel Künstner

**Affiliations:** 1 University of Lübeck, Lübeck, Germany

The submitted manuscript describes the genome assembly of the Four-spotted Footman moth ( *Lithosia quadra*). The genome was assembled using a single male individual, and the assembly comprises a total length of 456.27 MB. As males are the homogametic sex in *Lithosia quadra*, the assembly lacks the W sex chromosome. The mitochondrial genome is provided as well with the nuclear genome assembly.

 The methods section thoroughly describes sample collection, DNA extraction, sequencing (using PacBio HiFi long reads and Hi-C), assembly approach, and quality assessment. They used appropriate tools for genome assembly (Hifiasm), scaffolding (YaHS), and quality evaluation (BUSCO, Merqury.FK).

 The paper follows the standard format for Darwin Tree of Life genome notes, including appropriate figures showing assembly metrics, GC-coverage distributions, and Hi-C contact maps that validate the chromosomal-level scaffolding.

 Overall, the paper presents a technically sound chromosome-level genome assembly that meets the Earth BioGenome Project reference standard (6.C.Q66). The genome should be valuable for future studies on lepidopteran genomics and evolution.

 The data is available via the European Nucleotide Archive (Accession number PRJEB66748).

Are sufficient details of methods and materials provided to allow replication by others?

Yes

Is the rationale for creating the dataset(s) clearly described?

Yes

Are the datasets clearly presented in a useable and accessible format?

Yes

Are the protocols appropriate and is the work technically sound?

Yes

Reviewer Expertise:

Cancer genomics, Microbiome, Genome assembly

I confirm that I have read this submission and believe that I have an appropriate level of expertise to confirm that it is of an acceptable scientific standard.
